# T1 values and extracellular volume fraction in asymptomatic subjects: variations in left ventricular segments and correlation with cardiovascular risk factors

**DOI:** 10.1038/s41598-022-16696-0

**Published:** 2022-07-22

**Authors:** Moon Young Kim, Soo Jin Cho, Hae Jin Kim, Sung Mok Kim, Sang-Chol Lee, MunYoung Paek, Yeon Hyeon Choe

**Affiliations:** 1 Department of Radiology, Samsung Medical Center, Sungkyunkwan University School of Medicine, 81 Ilwon-ro, Gangnam-gu, Seoul, 06351 Republic of Korea; 2 Department of Radiology, Seoul Metropolitan Government Seoul National University Boramae Medical Center, 20, Boramae-ro 5-gil, Dongjak-gu, Seoul, Republic of Korea; 3 Health Promotion Center, Samsung Medical Center, Sungkyunkwan University School of Medicine, Seoul, Republic of Korea; 4 Department of Radiology, Eulji University Medical Center, Eulji University School of Medicine, Daejeon, Republic of Korea; 5 Cardiovascular Imaging Center, Samsung Medical Center, Heart Vascular and Stroke Institute, Sungkyunkwan University School of Medicine, 81 Ilwon-ro, Gangnam-gu, Seoul, 06351 Republic of Korea; 6 Division of Cardiology, Department of Medicine, Samsung Medical Center, Sungkyunkwan University School of Medicine, Seoul, Republic of Korea; 7 Siemens Healthineers Ltd. Korea, 23 Chungjeong Ro, Seoul, Republic of Korea

**Keywords:** Cardiology, Diseases, Medical research

## Abstract

To evaluate variations in pre-contrast (preT1) and post-contrast (postT1) myocardial T1 values and extracellular volume fraction (ECV) according to left ventricular (LV) segments and to find correlations between them and cardiovascular risk factors. The 233 asymptomatic subjects (210 men, 23 women; aged 54.1 ± 6.0 years) underwent cardiac magnetic resonance imaging with preT1 and postT1 mapping on a 1.5-T scanner. T1 values and ECVs were evaluated according to LV segments, age, sex, and estimated glomerular filtration rate (eGFR). Based on the presence of hypertension (HTN) and diabetes mellitus (DM), subjects were subdivided into the control, HTN, DM, and HTN and DM (HTN-DM) groups. T1 values and ECV showed significant differences between septal and lateral segments at the mid-ventricular and basal levels (*p* ≤ 0.003). In subgroup analysis, the HTN-DM group showed a significantly higher ECV (0.260 ± 0.023) than the control (0.240 ± 0.021, *p* = 0.011) and HTN (0.241 ± 0.024, *p* = 0.041) groups. Overall postT1 and ECV of the LV had significant correlation with eGFR (r = 0.19, *p* = 0.038 for postT1; r =  − 0.23, *p* = 0.011 for ECV). Septal segments show higher preT1 and ECV but lower postT1 than lateral segments at the mid-ventricular and basal levels. ECV is significantly affected by HTN, DM, and eGFR, even in asymptomatic subjects.

## Introduction

Myocardial fibrosis (MF) is closely linked to various adverse cardiovascular events^1^. Recent research has found that “diffuse” fibrosis is more intimately correlated with poor outcomes, such as mortality or hospitalization, than “focal” fibrosis^2,3^. Even though the biopsy is the gold standard for confirming MF, native and post-contrast T1 mapping via the modified look-locker inversion recovery (MOLLI) sequence has become a foundational method for detecting MF and infiltrative processes^4,5^. Native T1 value and extracellular volume fraction (ECV) on cardiac magnetic resonance imaging (CMR) correlate well with the histological quantification of MF on a region-of-interest (ROI) basis^4,6–9^.

According to meta-analyses, pooled means of T1 values and ECV in normal subjects showed almost the same ECV and heterogeneous T1 values, and hypertension (HTN) yielded native T1 values and ECV similar to normal subjects^10,11^. However, they have not been evaluated segmentally, which could serve as reference values to evaluate localized myocardial abnormalities.

Recent reports have described a possible correlation between MF, demonstrated by elevated native T1 or ECV, and cardiovascular risk factors, such as HTN, diabetes mellitus (DM), and chronic renal disease^3,12–16^. As pressure overload of the left ventricle (LV) induces accelerated collagen synthesis and breakdown, MF is followed by left ventricular hypertrophy (LVH) in hypertensive heart disease^3,17^. One of the hallmarks of diabetic heart disease is diffuse interstitial MF, generated by increased collagen type III in the perimysium and perivascular regions^18^. Decreased glomerular filtration causes various organs to produce pro-fibrotic substances, leading to progress MF^15,19^. However, few studies have demonstrated alterations of T1 values and ECV in subjects with subclinical manifestations of those cardiovascular risk factors.

Therefore, this study aimed to report the variation in pre-contrast and post-contrast myocardial T1 values (preT1 and postT1, respectively) and ECV according to LV segments and evaluate any correlation between T1 values and cardiovascular risk factors in asymptomatic subjects.

## Results

The baseline characteristics and CMR LV measurements of the subjects and subgroups divided by their status of cardiovascular risk factors are shown in Table 1. The age of total subjects ranges from 40 to 80 years. Except for body mass index (BMI), blood pressure, and glycosylated hemoglobin (HbA1c), there was no statistically significant difference between subgroups with respect to each characteristic. The HTN and DM (HTN-DM) and HTN groups showed higher BMI, systolic blood pressure (SBP), diastolic blood pressure (DBP), and the DM and HTN-DM groups demonstrated higher HbA1c than the rest subgroups (*p* < 0.05).

### Segmental analysis of T1 values and ECV

**Figure 1:**
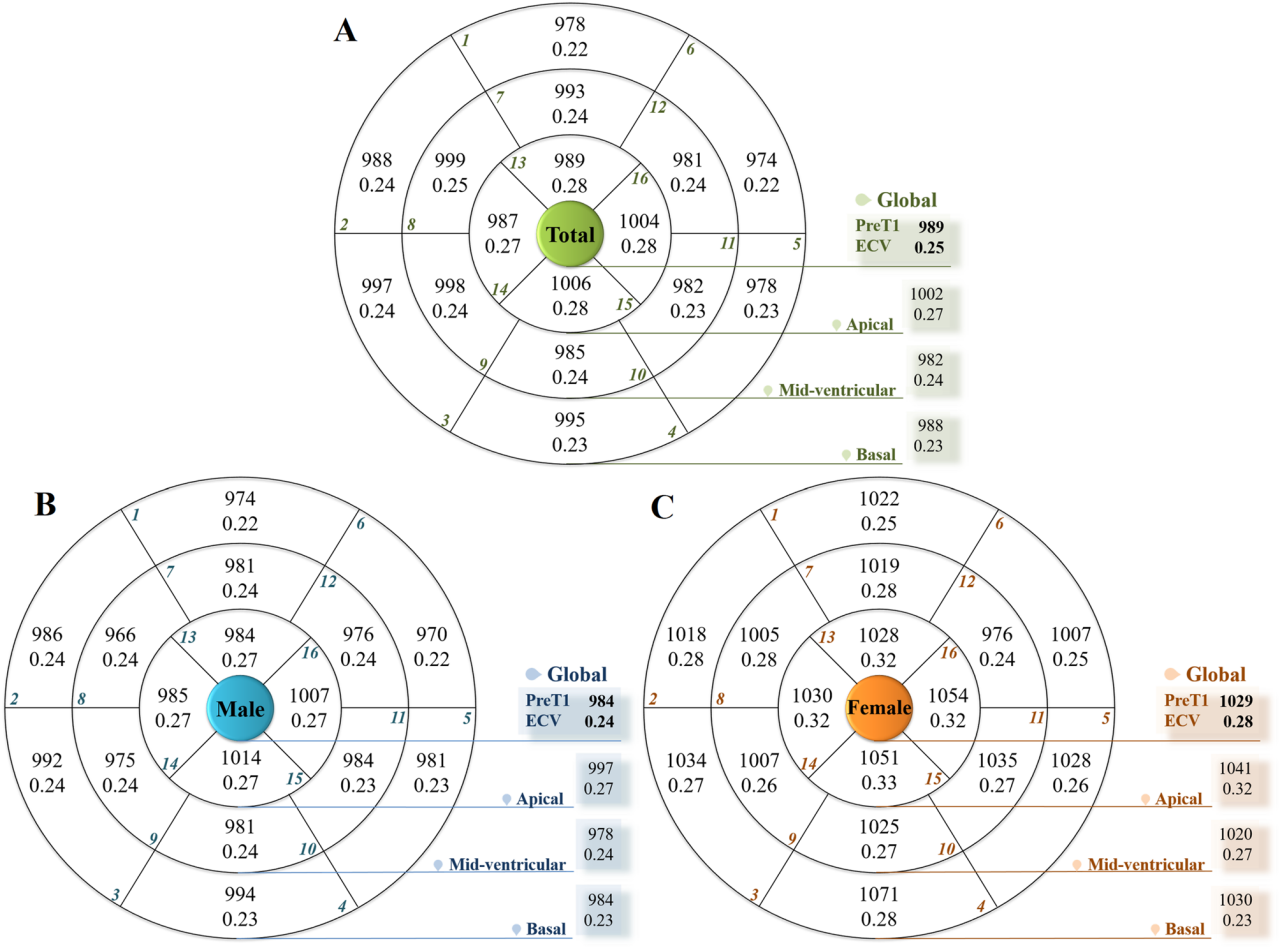
Mean segmental pre-contrast T1 value and mean extracellular volume fraction (ECV) of the left ventricle (LV). **(A)** Total study population. (**B**) Male. (**C)** Female. Pre-contrast T1 value and ECV were significantly higher in women than in men in global LV segments (*p* < 0.05) except apical inferior segment.

The mean T1 values and ECV with standard deviations for all 16 American Heart Association segments are shown in Fig. 1 and Supplementary Table S1. The overall intraclass correlation coefficient (ICC) for each segmental T1 value was 0.82 (*p* < 0.001). The ICC (0.64–0.80) for T1 value of each apical segment was significantly poorer than that (0.79–0.88) of each basal and mid-ventricular segment (*p* < 0.001, respectively). PreT1, postT1, and ECV of global LV myocardium were 989 ± 41 ms, 454 ± 38 ms, and 0.245 ± 0.024, respectively.

PreT1 and ECV revealed significantly higher values in the septal wall than in the lateral wall at the basal and mid-ventricular levels of the LV myocardium (*p* < 0.05, Table 2). However, postT1 values in the septal wall were significantly lower than those in the lateral wall at the basal and mid-ventricular levels of the LV myocardium (*p* < 0.05, Table 2).

### Comparisons of T1 values and ECV for age and sex

PreT1, postT1, and ECV of global LV myocardium in male were 984 ± 40 ms, 457 ± 36 ms, and 0.241 ± 0.021, respectively. Those in female were 1029 ± 25 ms, 443 ± 51 ms, and 0.280 ± 0.026, respectively. PreT1 and ECV were significantly higher in women than in men in all LV segments (*p* < 0.05, Fig. 1, Supplementary Table S1), except for the apical inferior segment (*p* = 0.053). However, postT1 showed no significant difference between male and female subjects (*p* ≥ 0.061).

There was no statistically significant difference in T1 values and ECV of the four age groups divided into quantiles (group 1 ≤ 50 years, n = 68; 50 years < group 2 ≤ 53 years, n = 58; 53 years < group 3 ≤ 56 years, n = 51; group 4 > 56 years, n = 56) (994 ± 46, 987 ± 40, 990 ± 45, and 991 ± 33 for preT1, *p* = 0.77; 451 ± 33, 454 ± 34, 446 ± 43, and 449 ± 43 for postT1, *p* = 0.95; 0.243 ± 0.029, 0.245 ± 0.027, 0.246 ± 0.021, and 0.245 ± 0.030 for ECV, *p* = 0.41, respectively) and no significant correlation (*p* > 0.46 for T1 values and ECV). In total population nor each sex group, there was no significant linear correlation between the age and T1 values or ECV (preT1, *p* = 0.58; postT1, p = 0.88; ECV, *p* = 0.45).

### Relations between T1 values and cardiovascular risk factors

**Figure 2:**
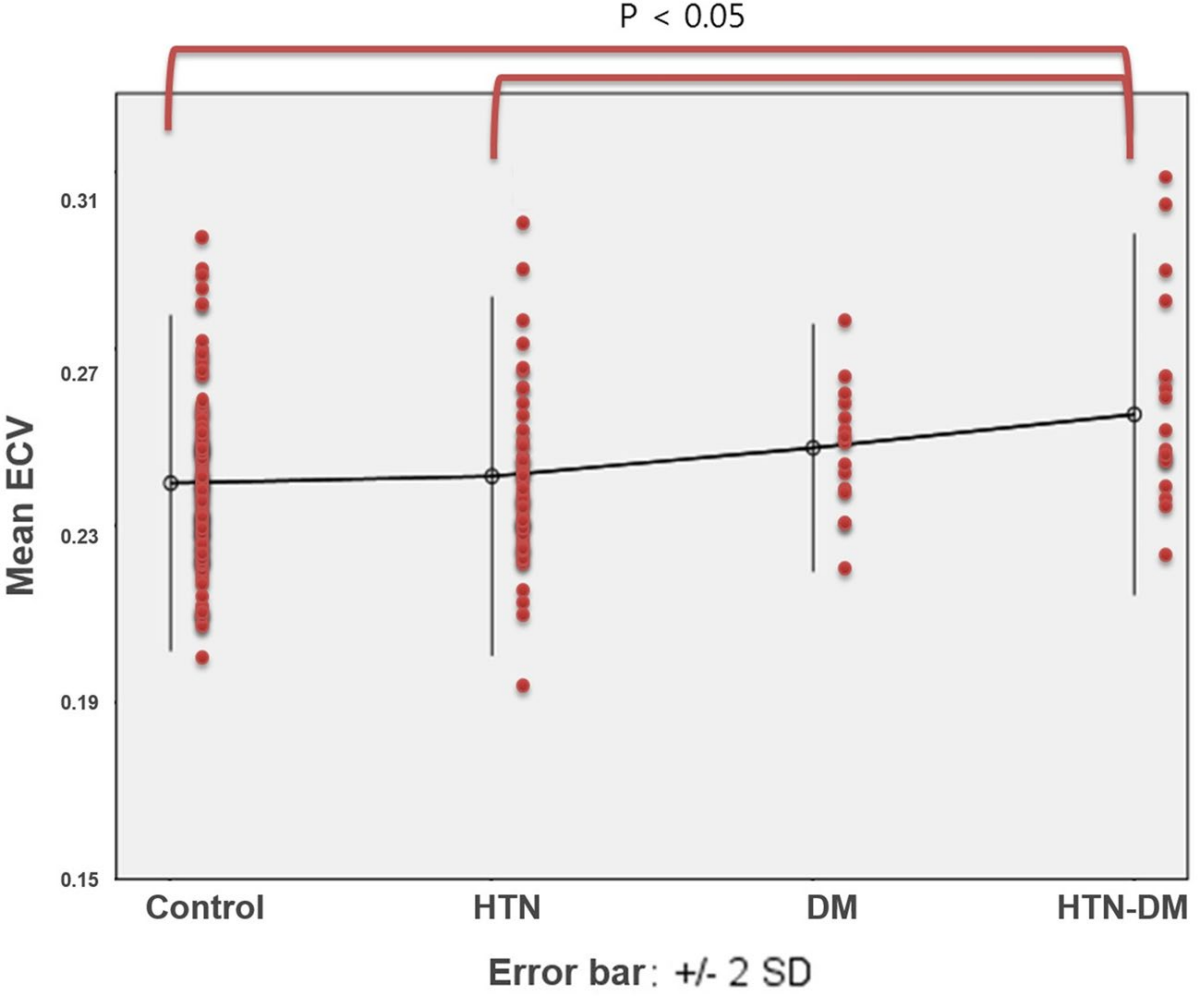
Relation of extracellular volume fraction (ECV) with hypertension (HTN) and diabetes mellitus (DM). HTN-DM group showed significantly higher ECV (0.260 ± 0.023) than control (0.240 ± 0.021, *p* = 0.011) and HTN groups (0.241 ± 0.024, *p* = 0.041). Error bars indicate 2 standard deviations (SDs) from average.

**Figure 3:**
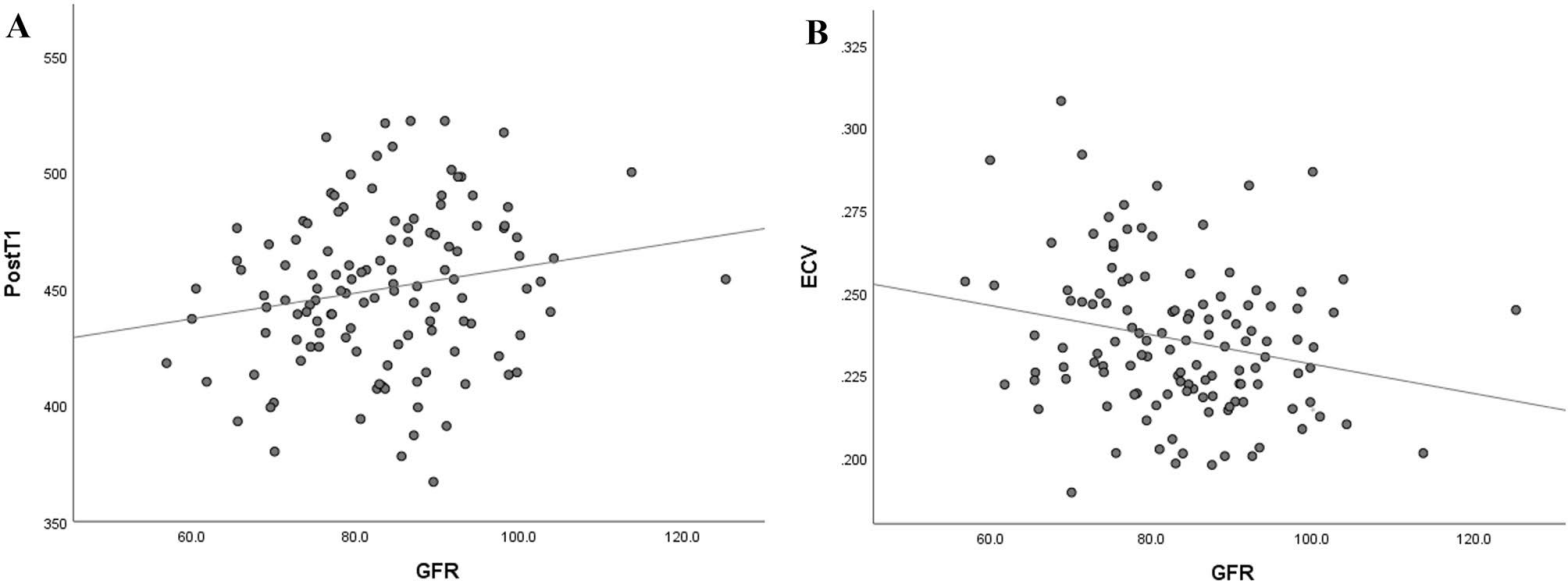
Relation of post-contrast T1 (PostT1) value and extracellular volume fraction (ECV) with glomerular filtration rate (GFR) in the control group. Overall PostT1 and ECV of left ventricle were proportional to GFR (A: r = 0.19, p = 0.038 for PostT1, B: r =  − 0.23, *p* = 0.011 for ECV).

A dot scatter plot of ECV with error bars indicating two standard deviations from the average reveals that the HTN-DM group showed significantly higher ECV (0.260 ± 0.023) than the control group (0.240 ± 0.021, p = 0.011) and the HTN group (0.241 ± 0.024, *p* = 0.041) (Fig. 2). There was a tendency for the ECV to increase among the subgroups in the following order: control, HTN, DM, and HTN-DM groups (*p* = 0.001). BMI nor dyslipidemia did not affect T1 values and ECVs statistically in the entire population and each subgroup (*p* > 0.05). PostT1 and ECV of the LV in the control group showed significant correlations to estimated glomerular filtration rate (eGFR) (r = 0.19 *p* = 0.038 for postT1; r =  − 0.23, *p* = 0.011 for ECV; Fig. 3). PreT1 demonstrated no significant correlation to eGFR (*p* = 0.100).

**Table 1:** Baseline characteristics and cardiac magnetic resonance measurements of study population.

Subgroup	Control group (*n* = 121, 51.9%)	HTN (*n* = 58, 24.9%)	DM (*n* = 25, 10.7%)	HTN-DM (*n* = 29, 12.4%)	*p*-value	Total (*n* = 233)
**Baseline characteristics**						
Age (years)	53.2 ± 4.98	55.8 ± 7.56	54.5 ± 5.56	54.5 ± 6.16	0.051	54.1 ± 5.97
Male	108 (87.8%)	53 (93.0%)	22 (91.7%)	27 (93.1%)	0.111	210 (90.1%)
BSA (m^2^)	1.83 ± 0.13	1.83 ± 0.15	1.79 ± 0.12	1.90 ± 0.17	0.076	1.83 ± 0.14
BMI (kg/m^2^)	24.3 ± 2.11	25.4 ± 2.53*^‡^	23.9 ± 2.54	26.7 ± 3.40*^‡^	< 0.001	24.8 ± 2.59
Heart rate (bpm)	66.0 ± 10.4	69.0 ± 12	68.5 ± 10.4	67.7 ± 11.3	0.330	67.2 ± 11.0
SBP (mmHg)	114 ± 14.3	125 ± 17.3*^‡^	114.6 ± 13.0	122 ± 17.4*^‡^	< 0.001	117 ± 16.0
DBP (mmHg)	76.0 ± 9.32	81.9 ± 10.2*^‡^	73.6 ± 9.41	79.0 ± 8.05^‡^	< 0.001	77.6 ± 9.76
Hematocrit (%)	44.1 ± 3.19	44.7 ± 2.99	44.9 ± 2.78	44.3 ± 2.73	0.773	44.3 ± 3.04
Hb (g/L)	15.0 ± 1.19	15.2 ± 1.12	15.3 ± 1.22	15.1 ± 1.06	0.656	15.1 ± 1.16
Hb A1c (%)	5.42 ± 0.22	5.30 ± 0.40	6.42 ± 0.53*^†^	6.21 ± 0.48*^†^	< 0.001	5.60 ± 0.36
eGFR (mL/min/1.73 m^2^)	83.5 ± 11.3	81.8 ± 15.6	83.6 ± 14.4	85.0 ± 10.2	0.720	83.3 ± 12.7
Dyslipidemia	70 (57.9%)	37 (63.8%)	11 (44.0%)	19 (65.5%)	0.269	137 (58.8%)
Hypertension	0	58 (100%)	0	29 (100%)		87 (37.3%)
Diabetes	0	0	25 (100%)	29 (100%)		54 (23.2%)
**Left ventricular measurements**						
EF (%)	65.75 ± 4.81	65.70 ± 5.79	65.25 ± 6.52	67.78 ± 6.07	0.378	65.9 ± 5.43
EDVi (mL/m^2^)	70.01 ± 10.22	67.79 ± 11.00	68.36 ± 8.24	67.68 ± 8.02	0.439	69.0 ± 9.99
ESVi (mL/m^2^)	24.16 ± 5.74	23.43 ± 6.25	23.90 ± 6.09	22.10 ± 5.85	0.391	23.7 ± 5.92
SVi (mL/m^2^)	59.21 ± 10.48	62.70 ± 11.78	54.82 ± 11.08	55.17 ± 10.98	0.097	59.1 ± 11.2
CI (L/min/ m^2^)	3.01 ± 0.44	3.01 ± 0.50	3.06 ± 0.28	3.22 ± 0.46	0.167	3.04 ± 0.45
ED MASSi (g/m^2^)	61.35 ± 10.89	65.45 ± 11.56	60.61 ± 9.16	63.24 ± 8.94	0.114	62.5 ± 10.8

*BMI* body mass index; *CI* cardiac index; *DM* diabetes mellitus; *DBP* diastolic blood pressure; *ED MASSi* indexed end-diastolic myocardial mass; *EDVi* indexed end-diastolic volume; *EF* ejection fraction; *ESVi* indexed end-systolic volume; *eGFR* estimated glomerular filtration rate; *Hb* hemoglobin; *HTN* hypertension; *SBP* systolic blood pressure; *SVi* indexed stroke volume.

Continuous values are presented as mean ± standard deviation.

The proportion of a numeric value is described in parenthesis.

* *p* < 0.05 versus control group.

^†^ *p* < 0.05 versus HTN group.

^‡^ *p* < 0.05 versus DM group.

**Table 2:** Comparing T1 values and ECVs between the septal and lateral walls.

	PreT1			PostT1			ECV		
	Septal	Lateral	p	Septal	Lateral	p	Septal	Lateral	p
Basal	992 ± 50	980 ± 52	0.001	458 ± 45	471 ± 40	< 0.001	0.240 ± 0.031	0.225 ± 0.026	< 0.001
Middle	998 ± 48	985 ± 61	0.003	454 ± 39	463 ± 40	< 0.001	0.242 ± 0.028	0.236 ± 0.035	< 0.001
Apical	989 ± 84	1011 ± 77	< 0.001	433 ± 50	430 ± 44	0.150	0.270 ± 0.048	0.278 ± 0.047	0.005
Mean	985 ± 48	992 ± 49	0.014	448 ± 41	454 ± 38	< 0.001	0.251 ± 0.029	0.246 ± 0.028	0.001

*ECV* extracellular volume fraction; *PreT1* pre-contrast myocardial T1 value; *PostT1* post-contrast myocardial T1 value.

Values are presented as mean ± standard deviation.

## Discussion

In this study, we presented reference values for native T1 relaxation time and ECV according to LV segments in asymptomatic subjects using a 1.5 T MR system. We found segmental variation in the myocardial tissue composition using CMR T1 mapping in asymptomatic subjects. The HTN-DM group showed a significantly higher ECV than the control and HTN groups. PostT1 and ECV of the LV showed significant correlations to eGFR.

MF is commonly associated with cardiac hypertrophy and failure and is associated with worsening ventricular systolic function, abnormal cardiac remodeling, and increased ventricular stiffness in animal models^20^. Although endocardial biopsy is the most specific procedure for measuring MF, it is invasive, and its sensitivity is low due to sampling errors^21^. CMR T1 mapping may serve as a noninvasive biopsy and collagen volume fraction (CVF) was associated with preT1 and ECV in an animal model of MF^22,23^.

In a pooled summary of 954 healthy subjects from the literature, 3-T showed longer T1 than 1.5-T with MOLLI variant sequences by approximately 100–200 ms. ECV of the healthy subjects was consistent among the studies (25–27%), irrespective of subject factors, sequences, vendors, and contrast types^11^. In a recent study, normal myocardial T1 reference ranges were reported as follows; at 3-T: 1129–1309 ms and at 1.5-T: 933–1020 ms (males) and 965–1054 ms (females), which tend to be similar to our findings^24^.

Our finding included segmental variations in T1 values and ECV, especially between septal and lateral segments. Motion during the T1 map acquisition may induce a poor T1 model fit and falsely deviated T1 values and ECV, because T1 maps are generated from a sequential series of images^25^. Lateral wall is more vulnerable to partial volume effects which may be a potential cause of the lower preT1 and ECV and the higher postT1 in the lateral segments.^26–29^. In addition, considering low reproducibility of apical T1 values, it may be reasonable to measure T1 values in basal to middle septal walls^28^.

Based on the result that preT1 and ECV were substantially higher in the septal wall than in the lateral wall at the basal and mid-ventricular levels of the LV myocardium, the thresholds of T1 and ECV for detecting MF or infiltrative disease at the lateral wall might be different from those at the septal wall. If the pathologic development and progression of myocardial disease are gradual, the very initial involvement of sarcoidosis or myocarditis at the lateral wall might be underestimated based on the reference values of the septal wall^30,31^. However, further investigations into this theory should be warranted as the T1 and ECV differences between septal and lateral walls might be subtler than those between normal and overt myocardial disease^32,33^.

On the other hand, in terms of LV anatomy or pathophysiology, there is no obvious consensus associated with variation of native T1 or ECV between septal and lateral segments. The cardiac skeleton and septal branches of the coronary artery may be partly accountable for increased T1 and ECV in the basal septum^34,35^. The myocardial perfusion difference between the septum and non-septal wall might also contribute to the variations in native T1^36,37^.Given that cardiac diseases with MF, such as hypertrophic cardiomyopathy, dilated cardiomyopathy, and pulmonary HTN, show increased native T1 and ECV, and late gadolinium enhancement (LGE), more frequently at the septal wall, it might be possible that the fibrotic remodeling of the LV starts earlier at the septum than the lateral wall^38–40^.

Sex hormones may influence myocardial structure and function^41,42^. Especially, a previous study demonstrated that estradiol attenuates myocardial hypertrophy^43^. Similar to prior literature, our observations of higher preT1 and ECV of females than those of males may suggest that sex hormones affect myocardial histology^26,44^. However, since the proportion of females in this study is small (9.9%), that result should be extrapolated carefully, considering other studies with balanced sex distribution.

There is inconsistency in association of age with T1 values and ECV in the published studies^26,27,44–47^. Rauhalamm et al.^26^ and Liu et al.^45^ demonstrated a linear correlation between age and T1 values and ECV. According to Rosmini et al., native myocardial T1 decreased slightly with age, while ECV did not change with age^47^. In our study, aging was not a significant factor affecting T1 values and ECV, which is consistent with a previous study by Dabir et al.^27^.

Our results showed that there was no significant difference in ECV between the HTN and control groups. According to previous reports, T1 mapping revealed increased diffuse MF in well-controlled HTN patients, but the increase was small and only occurred with LVH^48^. Our findings are consistent with the literature, since LVH was contained in the exclusion criteria of our study. Patients with HTN LVH had higher ECV and longer native T1 than HTN non-LVH and control subjects^5,48^. According to Venkatesh et al., in a study of 1,813 subjects who underwent CMR, HTN-induced remodeling was related to enhanced replacement and diffuse fibrosis^49^. In an animal study, the ECV and native T1 value of the myocardium in 18 hypertensive swine increased over three months, even though no LGE was found at any of the imaging times^23^.

According to Levelt et al., there were no significant differences in native myocardial T1 values and ECV between the patients with DM and the control subjects, indicating the absence of fibrosis in a similar context as our study^50^. Furthermore, our study showed HTN-DM group showed significantly high ECV than the control and HTN groups, which provides a clue about a synergetic effect on ventricular fibrosis of DM and HTN. DM appears to enhance fatty acid metabolism, decrease glucose oxidation, and change intracellular signaling, leading to cardiac structure and function alterations, based on prior research^51–53^. Ventricular fibrosis is a common structural and histological hallmark of DM, primarily owing to the deposition of collagen and advanced glycation end-products resulting from altered intracellular signaling and metabolic disturbances^54^. In a study of 135 patients with DM, ECV was significantly different from that of control subjects, while native T1 was not significantly different^55^. ECV values correlated with HbA1c levels in DM^56^. The group with HbA1c ≥ 7.0 had a significantly higher ECV value than the control subjects and the lower HbA1c group (HbA1c < 7.0). There was no statistically significant difference in the native T1 value and postT1 value among the three groups^56^. The lack of significance between the control and DM groups in ECV can be explained by Hb A1c below 7.2 in our research population.

In our study, in the control group, eGFR significantly correlated not with preT1 linearly but with postT1 proportionally and ECV inversely. This interesting finding suggests that postT1 may be associated with the degrees of renal clearance of contrast agent. Increased renal clearance of contrast agent could facilitate washout of contrast agent in myocardium and then increase myocardial postT1 values^57,58^. According to Edward et al., patients with chronic kidney disease had increased native T1 values and ECV compared with the control and hypertensive subjects^59^.

The limitations of this study include the retrospective cross-sectional study design, the relatively small number of subjects with DM (*n* = 25), DM and HTN (*n* = 29), and female subjects (*n* = 23). Especially, a relatively narrow range of age distribution concentrated in the 50 s resulting from the consecutive data collection could lower the reliability about the insignificant association between age and T1 values or ECV. Low ICCs for measurement of apical segments are ascribed to small ROIs and image inhomogeneity associated with motion.

In conclusion, there were significant differences between the T1 values and ECV of the septal and lateral walls at the mid-ventricular and basal levels in asymptomatic subjects. ECV is significantly affected by cardiovascular risk factors such as HTN, DM, and eGFR even before cardiovascular symptoms develop.

## Methods

This retrospective study was approved and the need for informed consent was waived considering the retrospective study nature by the Samsung Medical Center Institutional Review Board. All methods were carried out in accordance with relevant guidelines and regulations. The authors who have no relationships with the industry controlled the inclusion of all data and information in this article.

**Figure 4:**
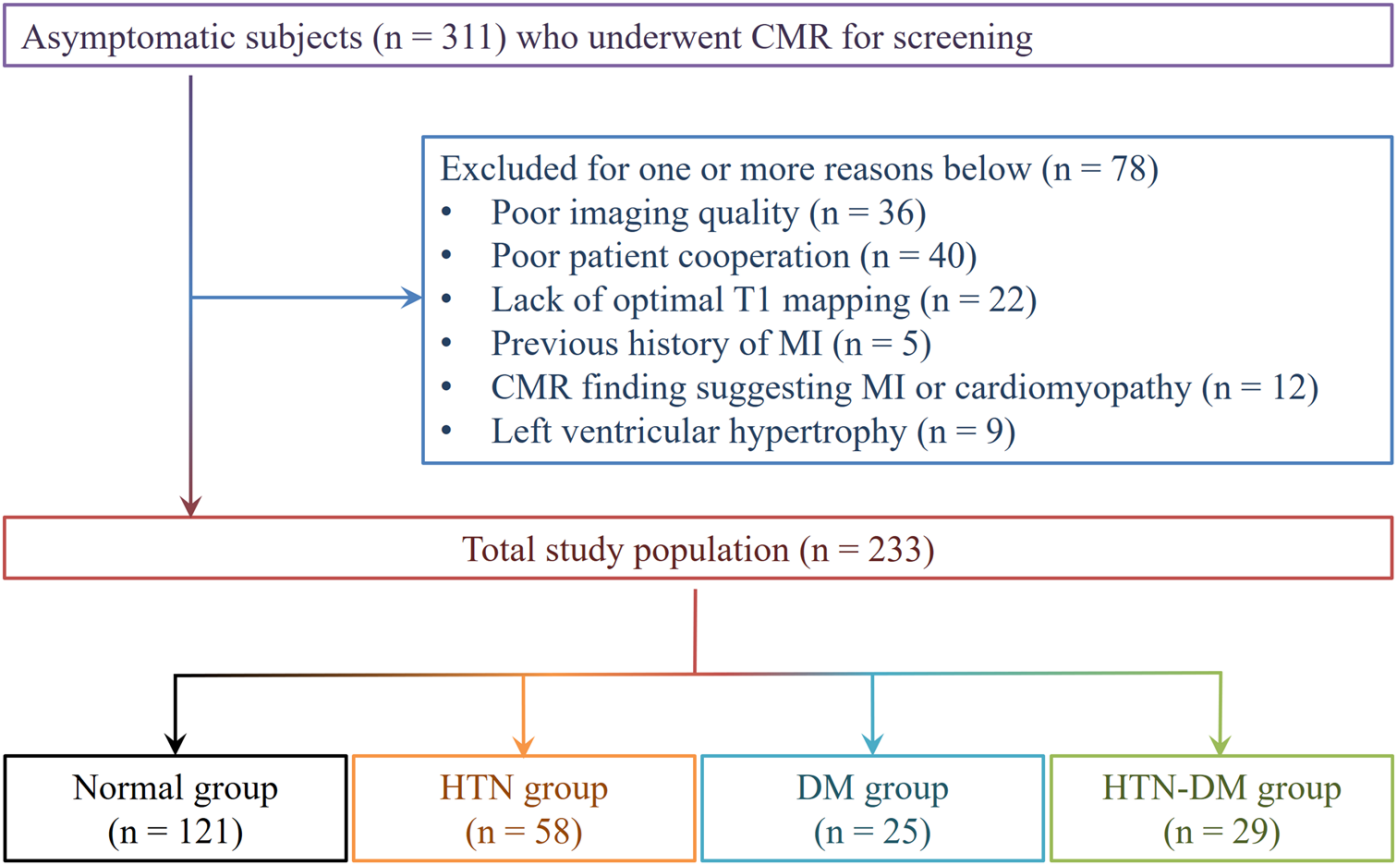
Flow chart of study population enrollment. The total study population was divided into four groups according to cardiovascular risk factors. The control group was defined as subjects who did not have hypertension (HTN) and diabetes mellitus (DM). Abbreviations: CMR, cardiac magnetic resonance imaging; MI, myocardial infarction.

We evaluated the records of consecutive asymptomatic subjects who underwent CMR for health screening at the Health Promotion Center of our institution between March 2014 and October 2015. We excluded subjects with one or more following criteria; poor imaging quality (*n* = 36) or cooperation (*n* = 40), lack of optimal T1 mapping (*n* = 22), and previous history of myocardial infarction (*n* = 5), localized T1 abnormality and LGE suspicious for myocardial infarction or cardiomyopathy (*n* = 12), LVH defined as LV wall thickness > 13 mm at least 1 segment or indexed end-diastolic mass > 91 g/m^2^ in males and > 77 g/m^2^ in females (*n* = 9)^60,61^. A total of 233 subjects were included (Fig. 4).

### CMR protocol

**Figure 5:**
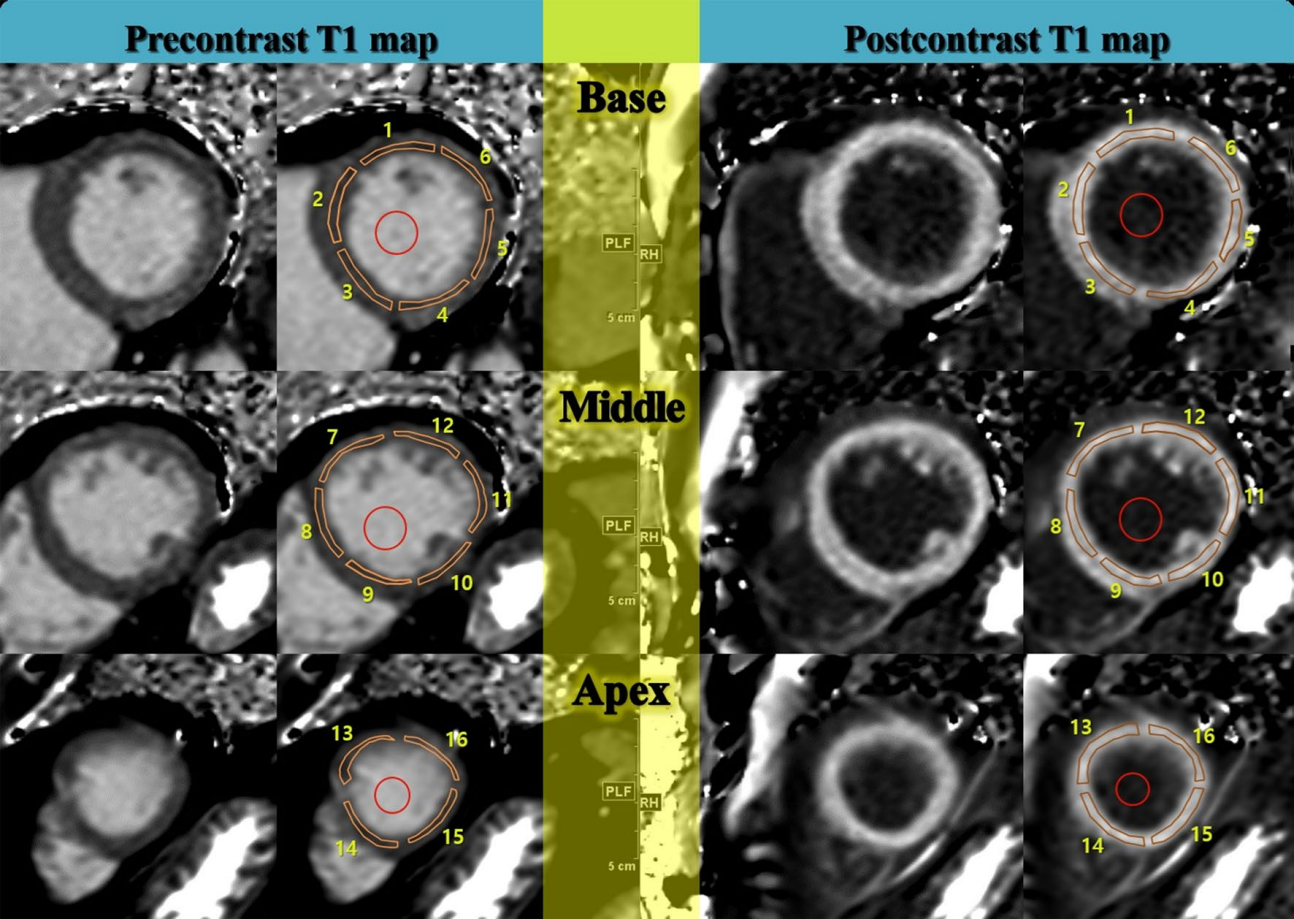
Short-axis slices of pre-contrast and post-contrast reconstructed T1 maps. The corresponding regions of interest on left ventricular segments and cavity blood (red circles) were drawn on T1 maps in a 54-year-old male in the control group.

All patients underwent CMR using a 1.5 T MR system (Magnetom Avanto, Siemens Healthineers) with a 32-channel phased-array receiver coil during repeated breath-holds. After localization, cine images of the LV mass were acquired using a steady-state free precession (SSFP) sequence with 8–10 contiguous short-axis slices to cover the entire LV with a slice thickness of 6 mm and a 4-mm gap. Standard delayed gadolinium-enhanced imaging was acquired using the phase-sensitive inversion recovery (PSIR) technique after injecting 0.15 mmol/kg gadobutrol (Gadovist; Bayer Healthcare) in 10–12 continuous short-axis images of 6 mm thickness with a 4-mm slice gap. Inversion delay times were typically 280–360 ms. Three short-axis images were acquired using the MOLLI sequence before and 15 min after administering gadolinium (Fig. 5).

Typical imaging parameters for MOLLI were as follows: two non-selective inversion pulses; number of acquisitions after each inversion pulse, 5, 3; number of recoveries before the second inversion pulse, 3; SSFP single-shot readout in mid-diastolic phase; field of view, 350 × 300 mm^2^; acquisition matrix, 192 × 150; slice thickness, 8 mm; flip angle, 35°; repetition time, 2.43 ms; echo time, 1.01 ms; bandwidth, 1085 Hz/pixel; minimum inversion time, 120 ms; inversion time increment, 80 ms; generalized autocalibrating partially parallel acquisition factor, 2.

### T1 mapping post-processing

The T1 maps were generated automatically by the scanner after the acquisition with MOLLI. To correct for residual cardiac and respiratory motion between individual inversion-recovery images, a non-rigid motion correction algorithm^62^ was used, and T1 values were calculated on a pixel-wise basis by performing a PSIR non-linear curve fitting using the three-parameter signal model.

### Image analysis

Image acquisition and analysis were done in a manner consistent with the consensus statement by the Society for Cardiovascular Magnetic Resonance (SCMR) endorsed by the European Association for Cardiovascular Imaging^4^. Global LV mass and functional parameters were analyzed using a dedicated software (Syngo.via; Siemens Healthineers). Image analysis of T1 maps was performed by two experienced cardiac radiologists (CMR experience of 7 years and 12 years, respectively) blinded to the clinical information. ROIs were drawn on 16 LV segments and blood pools manually (Fig. 5). Areas of nonspecific LGE at the right ventricular insertion points were excluded from measurement with ROIs. A circular ROI was placed in the LV cavity, avoiding papillary muscles, to assess the T1 value of blood. All original images were assessed for artifacts due to susceptibility and cardiac or respiratory motion. Each motion-corrected series was evaluated for correct image alignment and each map was evaluated as to whether the original images were transformed to an acceptable map^63^.

### ECV calculation

ECV was calculated using the following equations:

ΔR1 = 1/T1 time post-contrast—1/T1 time pre-contrast,

λ = ΔR1_myocardium_/ΔR1_blood_,

$$ {\text{ECV }} = \, \lambda * ({1} - {\text{Hct}}). $$

Hct refers to the hematocrit measured in venous blood sampled on the same day of the MRI scan acquisition ^4,64,65^.

### Clinical information

The clinical information of the subjects was obtained from medical records and laboratory findings on the day of CMR acquisition. BMI was calculated as weight (kg) divided by the square of the height (m^2^). SBP and DBP were measured after the subject had rested for at least 5 min. Blood samples were collected from the antecubital vein after overnight fasting. Total cholesterol, high-density lipoprotein, triglyceride (TG), fasting plasma glucose (FPG), and serum creatinine (Cr) levels were measured using enzymatic or colorimetric methods. The eGFR was calculated using a formula from the Modification of Diet in Renal Disease study: eGFR (mL/min/1.73 m^2^) = 186.3 × (Cr)—1.154 × (age)—0.203 × (0.742 for women)^66^. Body surface area (BSA) was calculated as follows: 0.20247 × height (m)^0.725^ × weight (kg)^0.425^ (Dubois formula). HTN was diagnosed when a person’s SBP in the office or clinic was ≥ 140 mmHg and/or their DBP was ≥ 90 mmHg following repeated examinations^67^. DM was diagnosed using the following criteria: occasional plasma glucose value of ≥ 200 mg/dL (≥ 11.1 mmol/L), FPG of ≥ 126 mg/dL (7.0 mmol/L) (fasting time 8–12 h), oral glucose tolerance test 2-h value in venous plasma ≥ 200 mg/dL (≥ 11.1 mmol/L), HbA1c ≥ 6.5% (≥ 48 mmol/mol Hb), and impaired fasting glucose for the fasting glucose range of 100–125 mg/dL (5.6 mmol/L- 6.9 mmol/L) in venous plasma^68^.

T1 values and ECV were compared among age groups and between sexes. According to their status of cardiovascular risk factors, subjects were divided into four subgroups: (1) HTN group, (2) DM group, (3) HTN-DM group, and (4) control group. The control group was defined as subjects who did not have HTN and DM. We evaluated whether there were any differences in T1 values and ECV between the subgroups.

### Statistical analysis

All analyses were conducted using SPSS 26 (IBM, Chicago, United states) and Rex (Version 3.0.3, RexSoft Inc., Seoul, Korea). Categorical variables were reported as percentages and continuous variables are presented as means with standard deviations. The differences between continuous variables were analyzed using the independent or paired *t*-test. Wilcoxon rank sum test and Mann Whitney U-test were used to analyze the differences between non-continuous variables. According to normality of distribution, analysis of variance or Kruskal–Wallis test was applied for multiple group comparison. For the trend analysis, Jonckheere-Terpstra test was adopted. The relationships between T1 values and cardiovascular risk factors were studied using simple correlation analysis with Pearson correlation coefficient (r). Inter-observer agreement was quantified using ICCs for all subjects. The threshold for statistical significance was set at *p* = 0.05 in the two-tailed tests.

## Supplementary Information

Supplementary Information.

## Data Availability

The datasets generated during and/or analyzed during the current study are available from the corresponding author on reasonable request.

## References

[1] Ambale-Venkatesh B (2019). Association of myocardial fibrosis and cardiovascular events: the multi-ethnic study of atherosclerosis. Eur. Heart J. Cardiovasc. Imaging.

[2] Yang EY (2019). Myocardial extracellular volume fraction adds prognostic information beyond myocardial replacement fibrosis. Circ. Cardiovasc. Imaging.

[3] Treibel TA (2020). Extracellular volume associates with outcomes more strongly than native or post-contrast myocardial T1. JACC Cardiovasc. Imaging.

[4] Messroghli DR (2017). Clinical recommendations for cardiovascular magnetic resonance mapping of T1, T2, T2* and extracellular volume: a consensus statement by the society for cardiovascular magnetic resonance (SCMR) endorsed by the European association for cardiovascular imaging (EACVI). J. Cardiovasc. Magn. Reson. Off. J. Soc. Cardiovasc. Magn. Reson..

[5] Kuruvilla S (2015). Increased extracellular volume and altered mechanics are associated with LVH in hypertensive heart disease, not hypertension alone. JACC Cardiovasc. Imaging.

[6] Bull S (2013). Human non-contrast T1 values and correlation with histology in diffuse fibrosis. Heart.

[7] Flett AS (2012). Diffuse myocardial fibrosis in severe aortic stenosis: an equilibrium contrast cardiovascular magnetic resonance study. Eur. Heart J. Cardiovasc. Imaging.

[8] Fontana M (2012). Comparison of T1 mapping techniques for ECV quantification Histological validation and reproducibility of shMOLLI versus multibreath-hold T1 quantification equilibrium contrast CMR. J. Cardiovasc. Magn. Reson. Off. J. Soc. Cardiovasc. Magn. Reson..

[9] Kim PK (2017). Myocardial T1 and T2 mapping: techniques and clinical applications. Korean J. Radiol..

[10] Gottbrecht M, Kramer CM, Salerno M (2019). Native T1 and extracellular volume measurements by cardiac MRI in healthy adults: a meta-analysis. Radiology.

[11] Vo HQ, Marwick TH, Negishi K (2020). Pooled summary of native T1 value and extracellular volume with MOLLI variant sequences in normal subjects and patients with cardiovascular disease. Int. J. Cardiovasc. Imaging.

[12] Salvador DB (2022). Diabetes and myocardial fibrosis; a systematic review and meta-analysis. JACC Cardiovasc. Imaging.

[13] Connelly KA, Sarak B (2022). Diabetes and myocardial fibrosis; is CMR the force leading to the rise of “scar wars”?*. JACC Cardiovasc. Imaging.

[14] Treibel TA (2015). Extracellular volume quantification in isolated hypertension - changes at the detectable limits?. J. Cardiovasc. Magn. Reson..

[15] Romero-González G, González A, López B, Ravassa S, Díez J (2020). Heart failure in chronic kidney disease: the emerging role of myocardial fibrosis. Nephrol. Dial. Transplant..

[16] Ng AC (2012). Association between diffuse myocardial fibrosis by cardiac magnetic resonance contrast-enhanced T(1) mapping and subclinical myocardial dysfunction in diabetic patients: a pilot study. Circ. Cardiovasc. Imaging.

[17] Müller-Brunotte R (2007). Myocardial fibrosis and diastolic dysfunction in patients with hypertension: results from the Swedish Irbesartan left ventricular hypertrophy investigation versus atenolol (SILVHIA). J. Hypertens..

[18] Shimizu M (1993). Collagen remodelling in myocardia of patients with diabetes. J. Clin. Pathol..

[19] Matsushita K (2010). Association of estimated glomerular filtration rate and albuminuria with all-cause and cardiovascular mortality in general population cohorts: a collaborative meta-analysis. Lancet.

[20] Kwiecinski J (2020). Progression and regression of left ventricular hypertrophy and myocardial fibrosis in a mouse model of hypertension and concomitant cardiomyopathy. J. Cardiovasc. Magn. Reson. Off. J. Soc. Cardiovasc. Magn. Reson..

[21] From AM, Maleszewski JJ, Rihal CS (2011). Current status of endomyocardial biopsy. Mayo Clin. Proc..

[22] Zeng M (2016). Histological validation of cardiac magnetic resonance T(1) mapping for detecting diffuse myocardial fibrosis in diabetic rabbits. J. Magn. Reson. Imaging.

[23] Zhuang B (2020). Detection of myocardial fibrosis and left ventricular dysfunction with cardiac MRI in a hypertensive swine model. Radiol. Cardiothorac. Imaging.

[24] Higgins DM, Keeble C, Juli C, Dawson DK, Waterton JC (2019). Reference range determination for imaging biomarkers: myocardial T(1). J. Magn. Reson. Imaging.

[25] Ferreira VM (2012). Non-contrast T1-mapping detects acute myocardial edema with high diagnostic accuracy: a comparison to T2-weighted cardiovascular magnetic resonance. J. Cardiovasc. Magn. Reson. Off. J. Soc. Cardiovasc. Magn. Reson..

[26] Rauhalammi SM (2016). Native myocardial longitudinal (T1) relaxation time: regional, age, and sex associations in the healthy adult heart. J. Magn. Reson. Imaging.

[27] Dabir D (2014). Reference values for healthy human myocardium using a T1 mapping methodology: results from the International T1 Multicenter cardiovascular magnetic resonance study. J. Cardiovasc. Magn. Reson..

[28] Rogers T (2013). Standardization of T1 measurements with MOLLI in differentiation between health and disease–the ConSept study. J. Cardiovasc. Magn. Reson. Off. J. Soc. Cardiovasc. Magn. Reson..

[29] Rogers T, Puntmann VO (2014). T1 mapping - beware regional variations. Eur. Heart J. Cardiovasc. Imaging.

[30] Jia Z (2021). Detection of acute myocarditis using T1 and T2 mapping cardiovascular magnetic resonance: a systematic review and meta-analysis. J. Appl. Clin. Med. Phys..

[31] Pour-Ghaz I (2021). Cardiac sarcoidosis: pathophysiology, diagnosis, and management. Hearts.

[32] Greulich S (2016). Comprehensive cardiovascular magnetic resonance assessment in patients with sarcoidosis and preserved left ventricular ejection fraction. Circ. Cardiovasc. Imaging.

[33] Thongsongsang R, Songsangjinda T, Tanapibunpon P, Krittayaphong R (2021). Native T1 mapping and extracellular volume fraction for differentiation of myocardial diseases from normal CMR controls in routine clinical practice. BMC Cardiovasc. Disord..

[34] Saremi F (2017). Fibrous skeleton of the heart: anatomic overview and evaluation of pathologic conditions with CT and MR imaging. Radiographics.

[35] Nakamura M (2020). What is the mid-wall linear high intensity "lesion" on cardiovascular magnetic resonance late gadolinium enhancement?. J. Cardiovasc. Magn. Reson. Off. J. Soc. Cardiovasc. Magn. Reson..

[36] Muehling OM (2004). Regional heterogeneity of myocardial perfusion in healthy human myocardium: assessment with magnetic resonance perfusion imaging. J. Cardiovasc. Magn. Reson..

[37] Kawel N (2012). T1 mapping of the myocardium: intra-individual assessment of the effect of field strength, cardiac cycle and variation by myocardial region. J. Cardiovasc. Magn. Reson. Off. J. Soc. Cardiovasc. Magn. Reson..

[38] Shehata ML (2011). Myocardial delayed enhancement in pulmonary hypertension: pulmonary hemodynamics, right ventricular function, and remodeling. AJR Am. J. Roentgenol..

[39] Spruijt OA (2016). Increased native T1-values at the interventricular insertion regions in precapillary pulmonary hypertension. Int. J. Cardiovasc. Imaging.

[40] Dong Y (2018). Age and gender impact the measurement of myocardial interstitial fibrosis in a healthy adult chinese population: a cardiac magnetic resonance study. Front. Physiol..

[41] Regitz-Zagrosek V (2006). Therapeutic implications of the gender-specific aspects of cardiovascular disease. Nat. Rev. Drug Discov..

[42] Mendelsohn ME, Karas RH (2005). Molecular and cellular basis of cardiovascular gender differences. Science.

[43] van Eickels M (2001). 17beta-estradiol attenuates the development of pressure-overload hypertrophy. Circulation.

[44] Piechnik SK (2013). Normal variation of magnetic resonance T1 relaxation times in the human population at 15 T using ShMOLLI. J. Cardiovasc. Magn. Reson. Off. J. Soc. Cardiovasc. Magn. Reson..

[45] Liu CY (2013). Evaluation of age-related interstitial myocardial fibrosis with cardiac magnetic resonance contrast-enhanced T1 mapping: MESA (Multi-Ethnic Study of Atherosclerosis). J. Am. Coll. Cardiol..

[46] Piechnik SK (2012). Age and gender dependence of pre-contrast T1-relaxation times in normal human myocardium at 1.5T using ShMOLLI. J. Cardiovasc. Magn. Reson..

[47] Rosmini S (2018). Myocardial native T1 and extracellular volume with healthy ageing and gender. Eur. Heart J. Cardiovasc. Imaging.

[48] Treibel TA (2015). Extracellular volume quantification in isolated hypertension - changes at the detectable limits?. J. Cardiovasc. Magn. Reson. Off. J. Soc. Cardiovasc. Magn. Reson..

[49] Ambale Venkatesh, B. *et al.* Association of longitudinal changes in left ventricular structure and function with myocardial fibrosis: the Multi-Ethnic Study of Atherosclerosis study. *Hypertension (Dallas, Tex. : 1979)* **64**, 508–515, 10.1161/HYPERTENSIONAHA.114.03697 (2014). 10.1161/HYPERTENSIONAHA.114.03697 PMC4134415 24914198

[50] Levelt E (2016). Relationship between left ventricular structural and metabolic remodeling in type 2 diabetes. Diabetes.

[51] Miki T, Yuda S, Kouzu H, Miura T (2013). Diabetic cardiomyopathy: pathophysiology and clinical features. Heart Fail Rev..

[52] Loncarevic B, Trifunovic D, Soldatovic I, Vujisic-Tesic B (2016). Silent diabetic cardiomyopathy in everyday practice: a clinical and echocardiographic study. BMC Cardiovasc. Disord..

[53] Boudina S, Abel ED (2010). Diabetic cardiomyopathy, causes and effects. Rev. Endocr. Metab. Disord..

[54] van Heerebeek L (2008). Diastolic stiffness of the failing diabetic heart: importance of fibrosis, advanced glycation end products, and myocyte resting tension. Circulation.

[55] Jiang L (2020). The combined effects of cardiac geometry, microcirculation, and tissue characteristics on cardiac systolic and diastolic function in subclinical diabetes mellitus-related cardiomyopathy. Int. J. Cardiol..

[56] Gao Y (2019). Evaluation of myocardial fibrosis in diabetes with cardiac magnetic resonance T1-mapping: correlation with the high-level hemoglobin A1c. Diabetes Res. Clin. Pract..

[57] Gai N (2011). T1 mapping of the gadolinium-enhanced myocardium: adjustment for factors affecting interpatient comparison. Magn. Reson. Med..

[58] Boss A (2007). Quantitative assessment of glomerular filtration rate with MR gadolinium slope clearance measurements: a phase I trial. Radiology.

[59] Edwards NC (2015). Diffuse interstitial fibrosis and myocardial dysfunction in early chronic kidney disease. Am. J. Cardiol..

[60] Maceira AM, Prasad SK, Khan M, Pennell DJ (2006). Normalized left ventricular systolic and diastolic function by steady state free precession cardiovascular magnetic resonance. J. Cardiovasc. Magn. Reson. Off. J. Soc. Cardiovasc. Magn. Reson..

[61] Massera D (2019). Prevalence of unexplained left ventricular hypertrophy by cardiac magnetic resonance imaging in MESA. J Am Heart Assoc.

[62] Xue H (2012). Motion correction for myocardial T1 mapping using image registration with synthetic image estimation. Magn. Reson. Med..

[63] Roller FC, Harth S, Schneider C, Krombach GA (2015). T1, T2 mapping and extracellular volume fraction (ECV): application, value and further perspectives in myocardial inflammation and cardiomyopathies. Rofo.

[64] Ugander M (2012). Extracellular volume imaging by magnetic resonance imaging provides insights into overt and sub-clinical myocardial pathology. Eur. Heart J..

[65] Pattanayak P, Bleumke DA (2015). Tissue characterization of the myocardium: state of the art characterization by magnetic resonance and computed tomography imaging. Radiol. Clin. North Am..

[66] Using standardized serum creatinine values in the modification of diet in renal disease study equation for estimating glomerular filtration rate. *Annal. Intern. Med.* **145**, 247–254, 10.7326/0003-4819-145-4-200608150-00004 %m 16908915 (2006). 10.7326/0003-4819-145-4-200608150-00004 16908915

[67] Unger T (2020). 2020 international society of hypertension global hypertension practice guidelines. Hypertension.

[68] Petersmann A (2019). Definition, classification and diagnosis of diabetes mellitus. Exp. Clin. Endocrinol Diabetes.

