# Supplementary material for: T1 values and extracellular volume fraction in asymptomatic subjects: variations in left ventricular segments and correlation with cardiovascular risk factors
Source: Sci Rep. 2022 Jul 22;12:12544. doi: 10.1038/s41598-022-16696-0 (PMC9307856; doi:10.1038/s41598-022-16696-0)
Supplement: Supplementary file 1 — Supplementary Information. [file 41598_2022_16696_MOESM1_ESM.pdf]

T1 Values and Extracellular Volume Fraction in Asymptomatic Subjects: Variations in Left Ventricular Segments and Correlation with Cardiovascular Risk Factors

Moon Young Kim, MD, Soo Jin Cho, MD, Hae Jin Kim, MD, PhD, Sung Mok Kim, MD, Sang-Chol Lee, MD, PhD, MunYoung Paek, PhD, Yeon Hyeon Choe, MD, PhD

Corresponding author: Yeon Hyeon Choe, MD, PhD
Department of Radiology and Cardiovascular Imaging Center, Heart Vascular and Stroke Institute, Samsung Medical Center, Sungkyunkwan University School of Medicine,
E-mail: yhchoe@skku.edu.

Supplementary table S1. Segmental T1 values and extracellular volume fraction (ECV) of the left ventricle.

	PreT1				***PostT1***				***ECV***			
LV	Total	Male	female	p value	Total	Male	female	p value	Total	Male	female	p value
Global	989±41	984±40	1029±25	0.000*	454±38	457±36	443±51	0.263	0.245± 0.024	0.241± 0.021	0.280± 0.026	0.000*
Basal	988±41	984±39	1030±41	0.000*	464±39	465±37	453 ± 51	0.284	0.233± 0.022	0.230± 0.019	0.266± 0.023	0.000*
Middle	982±46	978±46	1020±36	0.000*	454±39	455±38	449 ± 48	0.502	0.242± 0.024	0.239± 0.022	0.270± 0.027	0.000*
apical	1002±61	997±62	1041±45	0.001*	431±45	433±43	414 ± 56	0.144	0.274± 0.042	0.269± 0.040	0.320± 0.034	0.000*
1	978±64	974 ± 65	1022±57	0.001*	469±41	473±39	463 ± 61	0.428	0.223± 0.032	0.220± 0.030	0.254± 0.026	0.000*
2	988±55	986±51	1018±65	0.005*	452±43	459±54	441 ± 49	0.143	0.241± 0.040	0.238± 0.037	0.277± 0.049	0.000*
3	997±69	992±69	1034±67	0.004*	457±47	460±45	456 ± 57	0.661	0.239± 0.033	0.236± 0.032	0.265± 0.038	0.000*
4	995±60	994±87	1071±79	0.000*	458±57	463±47	449 ± 54	0.182	0.238± 0.035	0.233± 0.033	0.278± 0.034	0.000*
5	978±61	981±60	1028±60	0.000*	465±44	468±42	459 ± 50	0.377	0.230± 0.035	0.27± 0.034	0.259± 0.030	0.000*
6	974±68	970±68	1007±42	0.001*	473±44	476±40	464 ± 65	0.402	0.220± 0.032	0.216± 0.030	0.250± 0.032	0.000*

7	993±79	981±78	1019± 82	0.030*	456±44	460±42	443 ± 53	0.075	0.240± 0.037	0.236± 0.034	0.276± 0.045	0.000*
8	999±55	966±51	1005±62	0.001*	446±39	450±37	440 ± 57	0.439	0.246± 0.029	0.243± 0.027	0.276± 0.030	0.000*
9	998±55	975±53	1007±55	0.007*	456±42	459±40	459 ± 55	0.963	0.239± 0.033	0.237± 0.032	0.256± 0.032	0.001*
10	985±72	981±70	1025±72	0.005*	457±47	460±47	455± 54	0.620	0.240± 0.042	0.237± 0.032	0.265± 0.038	0.000*
11	982±73	984±85	1035±72	0.001*	466±44	459±41	452±49	0.061	0.232± 0.038	0.228± 0.035	0.270± 0.039	0.000*
12	981±71	976±68	1035±69	0.000*	457±43	459±41	453±48	0.491	0.239± 0.038	0.236± 0.037	0.268± 0.040	0.000*
13	989±89	984±85	1028±81	0.019*	427±49	429±46	411±59	0.083	0.275± 0.044	0.270± 0.042	0.319± 0.033	0.000*
14	987±85	985±84	1030±79	0.016*	431±51	435±48	413±64	0.133	0.270± 0.048	0.265± 0.043	0.320± 0.064	0.001*
15	1006±90	1014±87	1051±76	0.053	434±56	438±54	411±65	0.126	0.275± 0.063	0.269± 0.061	0.327± 0.055	0.000*
16	1004±89	1007±77	1054±65	0.005*	427±45	431±43	418±57	0.316	0.278± 0.047	0.274± 0.045	0.316± 0.049	0.000*

Abbreviations: PreT1 and postT1, pre-contrast and post-contrast T1 values, respectively.

*, $p < 0.05$
